# Local Oxidative Stress Expansion through Endothelial Cells – A Key Role for Gap Junction Intercellular Communication

**DOI:** 10.1371/journal.pone.0041633

**Published:** 2012-07-23

**Authors:** Ilan Feine, Iddo Pinkas, Yoram Salomon, Avigdor Scherz

**Affiliations:** 1 Department of Plant Sciences, Weizmann Institute of Science, Rehovot, Israel; 2 Department of Biological Regulation, Weizmann Institute of Science, Rehovot, Israel; 3 Department of Chemical Research Support, Weizmann Institute of Science, Rehovot, Israel; University of Illinois at Chicago, United States of America

## Abstract

**Background:**

Major circulation pathologies are initiated by oxidative insult expansion from a few injured endothelial cells to distal sites; this possibly involves mechanisms that are important to understanding circulation physiology and designing therapeutic management of myocardial pathologies. We tested the hypothesis that a localized oxidative insult of endothelial cells (ECs) propagates through gap junction inter-cellular communication (GJIC).

**Methodology/Principal Findings:**

Cultures comprising the bEnd.3 cell line, that have been established and recognized as suitable for examining communication among ECs, were used to study the propagation of a localized oxidative insult to remote cells. Spatially confined near infrared illumination of parental or genetically modified bEnd.3 cultures, pretreated with the photosensitizer WST11, generated O_2_•^−^ and •OH radicals in the illuminated cells. Time-lapse fluorescence microscopy, utilizing various markers, and other methods, were used to monitor the response of non-illuminated bystander and remote cells. Functional GJIC among ECs was shown to be mandatory for oxidative insult propagation, comprising de-novo generation of reactive oxygen and nitrogen species (ROS and RNS, respectively), activation and nuclear translocation of c-Jun N-terminal kinase, followed by massive apoptosis in all bystander cells adjacent to the primarily injured ECs. The oxidative insult propagated through GJIC for many hours, over hundreds of microns from the primary photogeneration site. This wave is shown to be limited by intracellular ROS scavenging, chemical GJIC inhibition or genetic manipulation of connexin 43 (a key component of GJIC).

**Conclusion/Significance:**

Localized oxidative insults propagate through GJIC between ECs, while stimulating de-novo generation of ROS and RNS in bystander cells, thereby driving the insult's expansion.

## Introduction

ROS and RNS are highly potent chemical entities that play key roles in both normal and patho-physiological conditions. ROS are known as the spearheads of first line defense mechanisms against invading pathogens in the plant and animal kingdoms [1], [2]. Over the past two decades, numerous studies have shown that superoxide anion, hydrogen-peroxide and nitric oxide (O_2_•−, H_2_O_2_, and NO• respectively) are also important in regulating cell and tissue functions, including vascular cell growth [3], cell death [4], cell migration, vessel tone modulation, extra-cellular matrix modification [5], [6], and more. Evidently the level, lifetime, and biological context of ROS/RNS production, define their biological effect.

ROS and RNS are tightly linked to cardio-vascular functions under normal and patho-physiological circumstances [6]. Normally, their levels within the vascular lumen and tissues are well regulated both, enzymatically and non-enzymatically. However, under certain pathological conditions, immune and endothelial cells (ECs) produce large amounts of ROS and NO• [7], [8] instantaneously. These species may impair the delicate balance between ROS production and annihilation, inflicting deleterious effects that are often augmented by cross-talk between activated endothelial and immune cells. The subsequent vascular disorders, such as endothelial dysfunction and perfusion-arrest, underlie most of the cardio-vascular pathologies [7]. On the other hand ROS and RNS, generated by ionizing radiation or light activated sensitizers in the vascular lumen and/or ECs, function as the spearheads in therapeutic tumor ablation [9].

Pivotal to the cumulative damage of these pathologies and therapies, is the up and down propagation of the acute oxidative insult (OI) [10], [11], [12], [13], [14], [15], [16], termed “the bystander effect” [17]. Notably, the distal spreading of an OI presents a paradox; namely, the short life span of the involved radicals (microsecond and shorter times [18]) in the biological milieu does not allow for their migration (up to a few mm) from the primary site of insult to the observed boundaries of injury. Hence, any proposed mechanism should involve alternative elements of propagation [14] similar to, or different from those recently suggested for plant defense mechanisms [19], [20].

In recent years, several studies showed that gap junctions (GJs), composed of six transmembrane connexin (Cx) subunits arranged as cylindrical channels (∼1.5nm diameter) that connect adjacent cells, can facilitate the transfer of 1–3KDa molecules with some dependence on the cell type and physiological status [21], [22]. ECs express mostly Cx37, 40 and 43; the latter is considered an important component of GJIC in myocytes and ECs, and was previously detected in several endothelial cell lines *in-vitro*, as well as in locations of disturbed blood flow *in-vivo* [23], [24]. The involvement of this route of communication among neighboring cells was demonstrated in neuronal [25], [26] and myocardial ischemia–reperfusion injuries [22], as well as in liver injuries [27]. With regard to vascular therapeutic approaches, GJIC was shown to augment suicide gene-therapy [17] and is involved in the cancer cell bystander effects under ionizing radiation therapy [28]. However, to the best of our knowledge, no such study was conducted, thus far, with ECs and a locally generated oxidative insult.

In fact, most studies regarding ECs focused on their role in immune cell recruitment as a response to local injury. However, the function of ECs as vaso-regulators necessitates upstream and downstream signal transduction [29], as well as calcium ion mediated communication with the neighboring myocytes, in response to changes in oxygen and nitric oxide levels. GJIC, among ECs, has recently been recognized as being imperative in conducted vasodilatation [30], [31]. Hence, it is reasonable to hypothesize that GJIC plays an important role in mediating the OI propagation. Indeed, a recent study suggests that GJIC and H_2_O_2_ have a role in endothelial derived hyperpolarization in response to bradykinin [32], but the link between the two has not yet been clarified.

In this study, we propose novel means to experimentally induce confined OI (COI), in a small group of cultured ECs (bEnd.3 cell line), and to monitor the propagation of the insult to distal ECs within the monolayer. In brief, a computer steered laser beam is delivered through a photoactivation unit (spatial resolution ∼7µm), for 5 minutes, to a geometrically confined region within the EC monolayer culture, that has been pre-incubated with the bacteriochlorophyll based photosensitizer WST11 [33]. The photoactivated WST11 locally generates intracellular O_2_•− and •OH radicals [34]. ROS and RNS formation, apoptosis, calcium ion fluxes and stress related gene activation in bystander, non-illuminated cell populations is then followed, for up-to 24 hours post COI, by live cell time-lapse fluorescence microscopy (TLFM), as well as by other methods. Changes in the propagation distance and rate of the above events, in response to genetic manipulation of Cx43 [23] and chemical blockade of the endothelial GJs, suggest that GJIC provides a route for propagation of COI among ECs from the initial site of insult to remote cells, followed by cell death propagation.

## Materials and Methods

### Cell culture

PymT transformed mouse brain parental endothelial cell line (bEnd.3), and its clone bEnd.3-D2 [23] were kindly provided by Dr. Paolo Meda (University of Geneva Medical Center, Switzerland). Cells were cultured as monolayers in Dulbecco's modified Eagle's medium (DMEM) (Gibco/Invitrogen, Carlsbad, CA) supplemented with 10% fetal calf serum (FCS), 2mmol/L glutamine, 0.06 mg/ml penicillin, and 0.1 mg/ml streptomycin (Biological Industries, Bet Haemek, Israel). In order to maintain bEnd.3-D2 clone purity, cells were grown in the presence of 200U hygromycin B/ml (Calbiochem, San Diego, CA).

H5V mouse heart endothelial cells [35] were cultured as monolayers in DMEM: F-12 (1∶1), containing 10% FCS, 1% non-essential amino acids mixture, 2mmol/L glutamine, 0.06 mg/ml penicillin, and 0.1 mg/ml streptomycin (Biological Industries). Cells were transfected with the Cyto-Hyper plasmid (Evrogen, Moscow, Russia) [36] and lipofectamine (Gibco/Invitrogen), according to manufacturer's guidelines to create clones of H5V-Hyper cells. Stable clones were selected by the addition of 2mg/ml neomycin G418 (Calbiochem) and fluorescence activated cell sorting (FACS) (Table S1).

All cell lines were cultured at 37°C in 5% CO_2_ humidified atmosphere.

### Photosensitizer

WST11 [33] was provided by Steba Biotech Ltd. (Rehovot, Israel). Stock solution (2mmol/L) was prepared in PBS and further diluted to a final concentration of 20µmol/L in the culture medium. The Photosensitizer's concentration was determined spectroscopically in methanol at 748 nm, using ε = 1.2 10^5^ mol^−1^ cm^−1^. Stock solution was stored in the dark at −20°C until used.

### Imaging and photo-activation system

Our imaging system was constructed using an upright fluorescence Olympus BX61 microscope equipped with several auxiliary devices, all controlled by iQ Live Cell Imaging Software (Andor Technology, Belfast, N. Ireland). The light source for imaging was a Lambda DG-4 (Sutter Instruments, Novato, CA), equipped with a full spectrum xenon lamp. Excitation wavelength was controlled by the Lambda DG-4 filters, or by the microscope's motorized filter wheel. Emitted light from the sample was collected by a rear mounted Cascade 512B EM-CCD (Photometrics, Tucson, AZ), providing a 512X512 pixel matrix, 16µm per pixel. For WST11 photoactivation, a laser beam (755nm) was delivered using a Model 3900S Ti: Sapphire CW laser (Spectra-Physics, Irvine, CA), coupled to the system by a single mode fiber through an acousto-optic modulator to a fluorescence recovery after photobleaching and photo-activation (FRAPPA) unit (Andor Technology). The unit was aligned to the microscope's optical path, enabling computer steered high resolution temporal and spatial photoactivation. Olympus full water immersion objectives (UMPlanFlN/10X/0.3 N.A., and XLUMPlanFl X20/0.95 N.A.) and Zeiss (W Plan-Apochromat X63/1.0 N.A.) were used for imaging. An Olympus objective (PlanApon N 2X/0.08 N.A.), was used for photoactivation. All the filters used for imaging were purchased from Chroma (Bellows Falls, VT).

### Live cell Sample preparation for photoactivation and fluorescence imaging

Cell suspensions (20×10^4^) were cultured for 48h before the experiment to 80–90% confluence in a 35mm Petri dish with, or without coverslips (Nunc, NY, USA). The Petri dish was mounted on the microscope's heated temperature controlled plate (37°C). In order to avoid pH changes during time-lapse fluorescence microscopy the culture medium was replaced by CO_2_ independent medium (Gibco/Invitrogen), supplemented with 10% FCS and 2mmol/L glutamine, 1h before the addition of WST11 (20µmol/l). Fluorescence indicators that were added prior to, during or after incubation were used according to experimental requirements as indicated in Table S1. This procedure is further designated as standard pre-incubation protocol.

### Laser photo-activation and time-lapse fluorescence microscopy (TLFM) protocol

A 4×4mm field of a cell monolayer was randomly chosen using the X2 objective and a 300×300µm rectangle was selected for photoactivation by the FRAPPA unit. In experiments where physical cell/cell contact dependence was examined, linear scrapes were made (∼20µm wide) using a surgical blade causing the interruption of the monolayer mosaic. Pixel dwell time for photoactivation was set to 100msec and two repeats, providing total laser illumination time of 5min. Laser power and wavelength were set to 1.2mW (at the sample) and 755nm, respectively. Immediately after photoactivation, either TLFM was performed with the X10 or X20 water-immersion objectives, or the plate was placed in the culture incubator for later examination. Filter combination and camera parameters were determined according to experimental requirements, as indicated. Stacks and image recordings were analyzed off-line. Standard pre-incubation protocol, followed by laser photoactivation, is termed here and thereafter as confined oxidative insult (COI).

### Fluorescent viability probes

Post COI cell death was tracked by two common membrane integrity probes, propidium iodide (PI) and calcein-acetoxy methyl ester green (CaAMg, Molecular Probes). Exposure to CaAMg probe was terminated by three successive rinses with pre-warmed probe free culture medium, while PI was present in the culture medium throughout TLFM experiment. For more details see Table S1.

### Scrape load dye transfer (SLDT) experiment

To assess the extent of gap junctional intercellular communication (GJIC) in bEnd.3 cells, the scrape load dye transfer (SLDT) assay [37] was used. Briefly, the monolayers were incubated with carbenoxolone (CBX), a GJ uncoupler, or its' inactive analog, glycyrrhizic acid [38] (GZA, both 100µmol/L, Sigma, St. Louis, MO, USA), for 30 minutes in the culture incubator. The coverslips were then rinsed with PBS and the culture medium replaced by PBS containing 0.5mg/ml Lucifer yellow (LY) and 0.5mg/ml rhodamine–dextran conjugate (Sigma). Then, linear scrapes were gently made with a surgical blade and the monolayers were left in the dark for 5min to complete dye uptake and transfer. GJIC was measured by counting the number of cell rows to which LY migrated from the scratch. Rhodamine-dextran served as a control for the initial dye loading site.

### ROS detection and ROS scavenging

After COI, 2′,7′-dichlorofluorescin diacetate (DCFH-DA) was used to monitor intracellular ROS formation in the treated cultures, dihydroethidine (DHE) was used to specifically monitor superoxide in the treated cultures and the H5V-hyper clone (see “Cell culture” section) was used to monitor intra-cellular hydrogen-peroxide as detailed in Methods S1 and Table S1.

In order to determine the role of connexins in ROS propagation, monolayers were incubated with 100µmol/L CBX or GZA for 30 minutes prior to COI in the culture incubator. Three hours after COI, ROS propagation was analyzed with DCFH-DA.

To scavenge intracellular ROS, two antioxidants were used: N-acetyl-L-cysteine (NAC, final concentration 10mmol/L) and vitamin C (100µm/L) [39], both purchased from Sigma. The scavengers were added prior to, or after COI, according to experimental requirements.

### Detection of apoptotic markers

Following COI, caspase activity was monitored with the CaspACE FITC-VAD-FMK assay kit (Promega), as previously described [40] and phosphatidylserine translocation to the outer membrane was monitored with the Annexin-V-FITC assay [41]. For more details see Methods S1 and Table S1.

### Indirect immuno-fluorescence labeling of c-Jun N-terminal kinase (JNK), Cx43 and peroxynitrite

bEnd.3 monolayers were subjected to COI and placed back into the incubator for 3h. Next, the coverslips were washed three times with PBS, fixed with 4% paraformaldehyde for 20 minutes at 25°C, permeabilized for 5min with 0.1% tritonX-100 in PBS at 25°C, rinsed with PBS and blocked in 1% normal goat serum in PBS for 30min. For JNK: Monoclonal anti-diphosphorylated (activated) JNK (pJNK, Sigma J4750 [42]) and polyclonal rabbit anti-JNK (Sigma J4500, both diluted 1∶100 in blocking buffer) served as specific first antibodies and were incubated for one hour at 25°C. The coverslips were then rinsed in PBS and further incubated with secondary fluorescent Ab diluted 1∶200 (FITC conjugated donkey anti-mouse for pJNK and FITC conjugated goat anti-rabbit for gJNK, both purchased from Jackson Laboratories, Bar Harbor, ME), for one hour at 25°C, rinsed with PBS and counterstained with DAPI. Finally, the coverslips were mounted on glass slides, sealed with adhesive and kept refrigerated in the dark until examination by fluorescence microscopy as described in Table S1.

#### Connexin43 (Cx43) labeling

naïve untreated bEnd.3 cells fixation and staining procedures, were identical to those described above. Primary and secondary Ab comprised of monoclonal mouse anti-Cx43 (BD Cat. Number 610062) [43] diluted to 1∶100 and FITC conjugated donkey anti-mouse (Jackson Laboratories) diluted to 1∶200, respectively.

#### Peroxynitrite detection

bEnd.3 cells grown on cover slips were subjected to COI and placed back for 3h into the culture incubator. Next, coverslips were washed three times with PBS, placed for 1min in ethanol: acetic acid [95∶5] and incubated with anti-nitrotyrosine (10μg/ml, Millipore, Billerica, MA, Cat. Number 05–233), according to manufacturer's protocol [26]. Next, cells were incubated with secondary FITC conjugated donkey anti-mouse Ab (Jackson Laboratories) diluted to 1∶200 and counterstained with DAPI.

### Calcuim ion (Ca^+2^) detection

Ca^+2^ levels in bEnd.3 cells was determined by the fluorescent indicator Fluo-4 (Molecular Probes) [44]. Briefly, bEnd.3 monolayers were incubated with Fluo-4 AM (5µmol/L) for one hour at 37°C in HBSS, supplemented with 20 mmol/L Hepes and 2.5 mmol/L probenicid, 0.1% (w/v) BSA and 0.042% (v/v) pluronic acid F-127 (all from Sigma). Cells were then washed twice with pre-warmed, probe-free CO_2_-independent medium containing PI and subjected to COI. The cells were imaged by TLFM every five minutes, with identical imaging parameters for CaAMg detailed in Table S1.

### Data analysis

Image and stack analysis was performed by ImageJ freeware (NIH) [45] and CellProfiler [46]. Data analysis, statistical analysis and graphical representation were performed by MATLAB and Microsoft Excel. Student's t-test was used to determine statistical significance.

## Results

### Endothelial cell death propagation following confined oxidative insult (COI)

**Figure 1:**
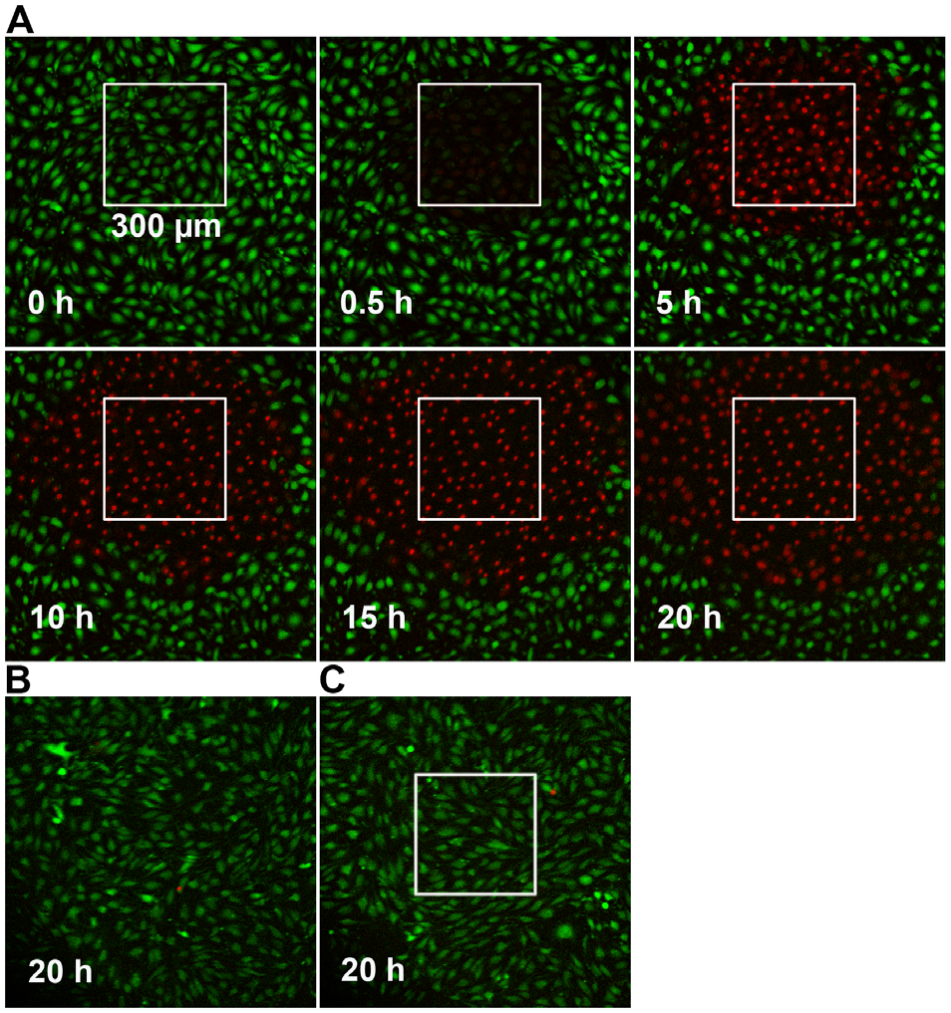
Propagation of cell death following COI. **A** – bEnd.3 monolayers were subjected to COI. Cell death was monitored by changes in the fluorescence of membrane viability probes, CaAMg and PI, as followed by TLFM. Dead cells are defined as CaAMg negative (green), PI positive (red). Time zero represents termination of COI. **B** – Dark control (with WST11, no illumination). **C** – Light control (Illumination, of same rectangle, no WST11). Both control images were taken at 20h. All other details are as described in the Materials and Methods section.

**Figure 2:**
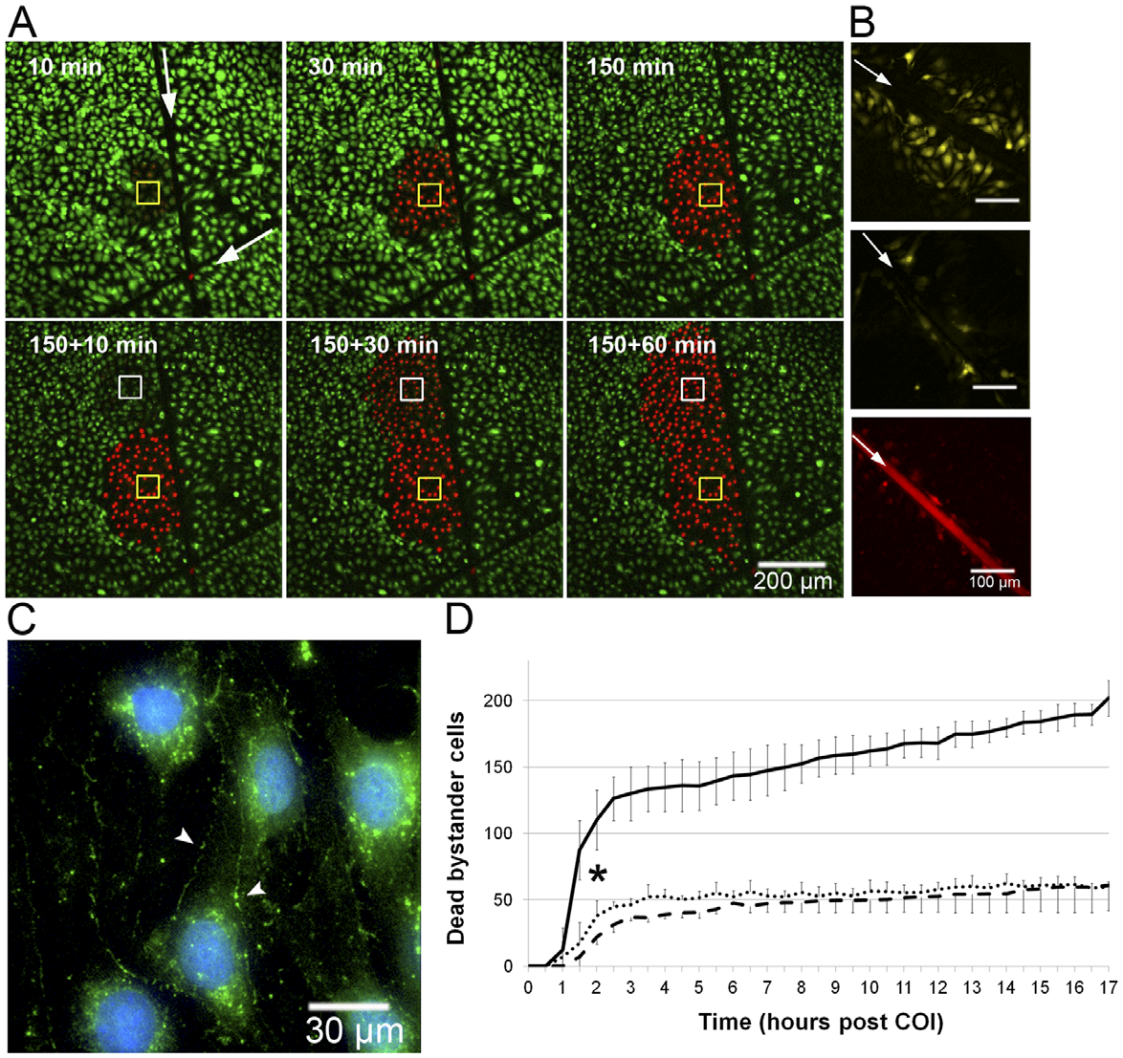
Death propagation among bystander cells requires cell-cell contact and connexins. **A** – Cell contact in bEnd.3 monolayer was impaired by surgical scalpel scratch (white arrows, scratch extends far beyond the field of view), and the culture was subjected to COI (60×60 µm, top left image, yellow rectangle) in one side of the scratch. Cell death (monitored as described in Fig. 1) propagates only in that side. Lower panel – an additional COI site was imposed at t = 150 min in the same side of the scratch (white rectangle). **B** – bEnd.3 cells were incubated with GZA (inactive analog, upper panel) or the GJ uncoupler CBX (middle panel). After 30min Lucifer yellow (LY, pseudo yellow) was added and SLDT was carried out (n = 12). White arrow marks the linear scratch. Rhodamine-dextran (red, lower panel) served as initial loading site control. **C** – bEnd.3 monolayers immunostained for Cx43, (pseudo-colored green) seen on cell membranes (arrow heads) and around the nuclei (counter stained with DAPI, pseudo-colored blue). Control experiments showed no non-specific binding of the secondary antibody (n = 3). **D** – The graph illustrates time dependent registration of PI^+^ cells beyond the COI rectangle in GZA treated (solid line), CBX treated (dashed line) and bEnd.3-D2 cells (dotted line) following COI. Values are displayed as means ± SD. * P<0.01 between the GZA and CBX/ bEnd.3-D2 experiments from t = >1.5h onwards (n = 4). All other details are described in Materials and Methods section.

Endothelial bEnd.3 cell monolayers were subjected to COI in a 300×300µm^2^ rectangular field. The spatial precision of light applied through the FRAPPA unit, previously shown by others [47], is also shown in Fig. S1, indicating no stray light delivery out of the assigned area of illumination. Figure 1A presents frames selected at the indicated time points from a 24h time-lapse video, initiated at the end of the COI (Video S1). Cell death within the first 30 minutes after the end of illumination is strictly limited to the COI domain, as indicated by loss of CaAMg fluorescence (green) and increased PI staining (red). The circle of PI stained dead cells (PI^+^) expanded across the surrounding non-illuminated bystander cells at a rate of ∼20µm/h, increasing the dead cell area from ∼9 to 50×10 µm^2^ by 20h. Dark control (WST11, no illumination) and light control (laser illumination, no WST11) showed no cell death during the 20h period (Fig. 1B, C). To examine the possibility that cell death progression requires cell-cell contact, the continuity of the monolayer was disrupted by scratching a 20µm wide groove (using a surgical scalpel) prior to COI. As can be seen, cell death progression stopped at the scratch and did not cross it, forming a semicircular area of dead cells limited only to the side of the COI rectangle (Fig. 2A); this continued in all other directions confirming that cell death propagation is cell-contact dependent. The need for cell contact to enable the propagation of death appears to contrast recent reports [48], [49] suggesting that cell death propagation, following photodynamic insult, is mediated by diffusible cytotoxic agents released into the culture medium by the dying cells.

### Gap junction intercellular communication (GJIC) among bEnd.3 endothelial cells is essential for the oxidative insult propagation – the role of connexin (Cx) 43

We hypothesized that GJIC connecting adjacent endothelial cells enables death propagation from primarily photoactivated bEnd.3 cells via non-illuminated bystander cells to remote sites. Connexin (Cx) 43 is a key component of GJIC in myocites and was previously reported to be upregulated in bEnd.3 ECs within areas of disturbed blood flow [23], [24] and other stress conditions, such as ionizing radiation [50]. To test the possible involvement of GJIC in the bEnd.3 endothelial cell death propagation, we first examined its' functionality, and then checked the effect of inhibiting Cx43 function on the impact of COI, as described below.

### Cx43 expression and function of GJIC of bEnd.3 cells

The scrape load dye transfer (SLDT) assay is a common and reliable method for analyzing and quantifying GJIC function [37]. Two fluorescent dyes were used: Lucifer yellow (LY), a low molecular weight dye (457Da) that can be transmitted by GJIC, and a rhodamine-dextran conjugate (70 KDa) that cannot pass through GJ due to its large size, which serves as an initial loading site indicator. A monolayer of bEnd.3 cells was injured by a linear scratch, as described above; both dyes were added to the culture medium and allowed to enter the scratched cells via their injured membrane. Figure 2B shows selected images of LY distribution among bEnd.3 cells after performing the SLDT procedure in the presence of carbenoxolone (CBX), a connexin inhibitor, or its inactive analog, glycyrrhizic acid (GZA) [38]. LY migrated through at least three rows of bystander cells in the presence of GZA (average propagation distance: 3.41±1.47 rows, Fig. 2B, upper panel). In contrast, CBX inhibited LY migration beyond the scratch (average propagation distance: 1.10±0.31 cell rows P<0.01, Fig. 2B lower panel). Rhodamine-Dextran, however, could not be detected beyond the injured cells on the scratch boundary, underscoring expression and functionality of GJIC in parental bEnd.3 endothelial cells. This conclusion was further substantiated by immunofluorescence staining of bEnd.3 cells (Fig. 2C), which revealed the presence of Cx43 in the peri-nuclear region (due to turnover, Cx43 has a half-life of 1–2h) and cell membranes, in agreement with previous reports [23].

### GJIC blockers inhibit death propagation among bEnd.3 cells

To test if GJIC controls death propagation in bEnd.3 endothelial cultures, COI was performed in the presence of CBX or GZA, to consider any nonspecific effects of the glycyrrhetinic acid. The fluorescence of the viability markers (PI and CaAMg) was monitored by TLFM and used to count numbers of dead bystander cells beyond the COI rectangle. Monolayers treated with GZA showed extensive bystander death propagation, however treatment with CBX significantly inhibited this effect (Fig. 2D).

In the bEnd.3-D2 clone, the expression of the Cx43-βGal protein elicits a dominant-negative fivefold inhibition effect in GJIC function [23], as determined by the dye coupling assays which were reproduced in our lab by the scrape load dye transfer assay (not shown). Therefore, any GJIC process mediated by Cx43 should be strongly attenuated in the mutant strain [23]. Figure 2D (dotted line) graphically shows that the dominant negative inhibition of GJIC strongly attenuated bystander cell death, to the extent seen upon CBX inhibition. In summary, the above results imply that GJIC and, Cx43 in particular, play a critical role in mediating bystander cell death propagation after COI.

### Intra-cellular ROS and RNS generation in bystander cells following COI

ROS and RNS have been recognized in the last few years as important mediators of acute oxidative insult and promoters of cell death and survival [51]. Hence, for further elucidation of the underlying features of bystander cell death we monitored the temporal evolution and propagation of these species in the treated cell cultures.

**Figure 3:**
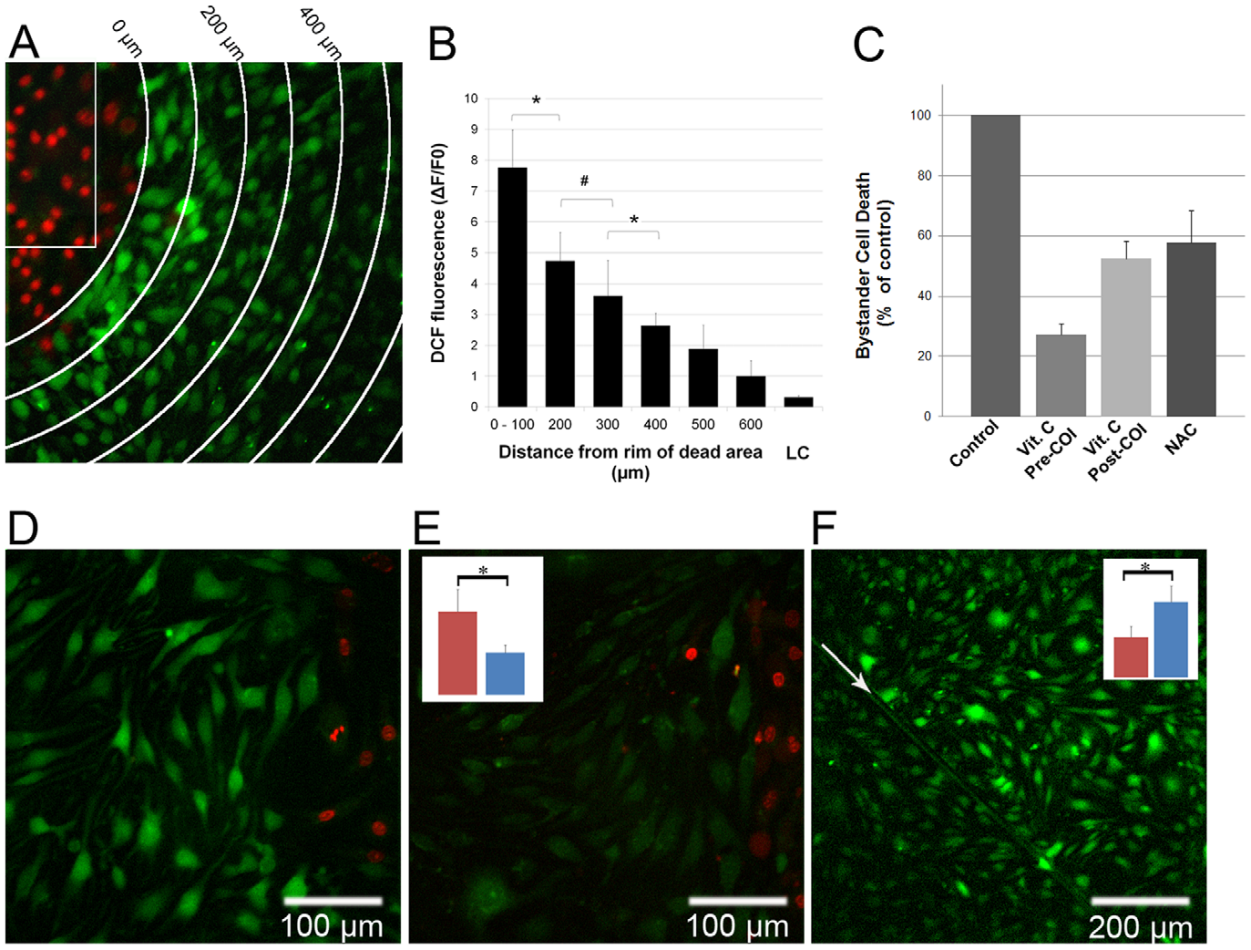
De-novo ROS generation in endothelial bystander cells and the effect of ROS scavengers and GJIC inhibition. bEnd.3 monolayers were incubated with DCFH-DA, rinsed with fresh culture medium and subjected to COI. After illumination the plates were placed in the incubator for 3h and then imaged for PI (pseudo-colored red) and DCF (ROS, pseudo-colored green) fluorescence. **A** – An overlay of two pseudo-colored captures, the white arches represent distance increments of 100µm from the COI (white rectangle), representative of n = 3. PI^+^ bystander cells on the top left corner are at 50–100µm from the rim of the COI. Adjacent to them on the first arch (0–100µm) are DCF positive, PI negative bystander cells. **B** – A plot of DCF fluorescence intensity (mean ± SD of five cells in three separate experiments). LC – light control, cells exposed to laser illumination, without WST11 incubation, imaged for DCF fluorescence 3h later. **C** – bEnd.3 monolayers were subjected to COI under the following conditions: Control: encompassing standard COI; Vit. C Pre-COI: encompassing incubation with 100µmol/L vitamin C for 1h, rinse and then subjected to COI; Vit. C Post-COI: encompassing the addition of 100µmol/L vitamin C immediately after COI; NAC: incubation with 10mmol/L NAC prior to COI. 3h after COI, cell death was determined by PI fluorescence. Notably, in all cases COI resulted in a complete cell death within the primary illuminated square. Values represent averaged percent of bystander cell death (mean ± SD of at least n = 3 separate experiments in each group) relative to the control. The control values are statistically significant higher than all treated groups (P<0.01). **D, E** – bEnd.3 monolayers were incubated with GZA (D) or CBX (E), respectively, subjected to COI and probed with DCFH-DA. Dead bystander cells (red) at a distance of 60–80µm from the rim of COI, are accompanied by PI^−^, DCF^+^ (representative of n = 3 experiments) in (D) but not in (E). The insert in (E) illustrates the mean DCF fluorescence of CBX compared to GZA treated cells (right and left colum respectively, n = 10 cells) at equal distances from the dead bystander cells. *- P<0.01 between the treatments. # - P<0.05. **F** – bEnd.3 monolayers were scratched by a surgical scalpel (arrow), underwent COI (near the upper right corner), and placed in the incubator for 10h. Intra-cellular ROS propagation (DCF^+^) is blocked at the scratch. Insert – right and left columns representing mean DCF fluorescence intensities in the photoactivation side and beyond the scratch, respectively). * P<0.01.

**Figure 4:**
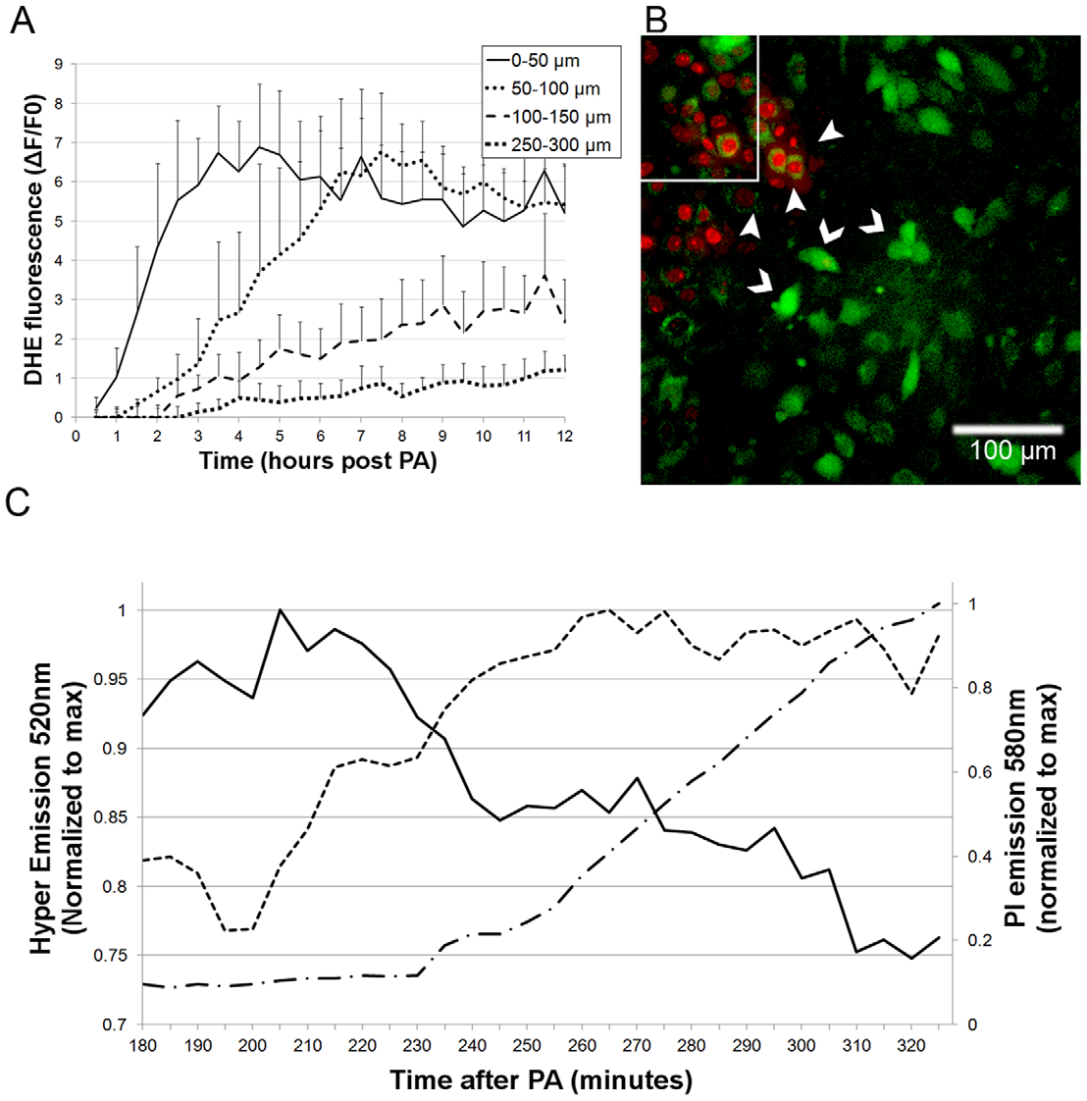
Superoxide anion (O_2_^•−^) and H_2_O_2_ detection in bystander cells following localized oxidative insult. **A** – Time dependent DHE fluorescence increase (superoxide generation) in bEnd.3 cells at four selected distances from the COI boundary, within the monolayers (n = 10 cells, ± SD). **B, C** – H5V-Hyper monolayers supplemented with PI and 100UI/ml catalase, were subjected to COI and imaged. **B** – An overlay of Hyper protein fluorescence (H_2_O_2_ elevation, intense green, 520nm emission, chevrons) and PI^+^ (red, at a distant of 30–40µm from the white rectangle COI rim, arrowheads) emissions acquired 3h after the COI. **C** – Time dependent fluorescence intensity of Hyper-low (solid line), Hyper-high (dashed line) and PI (dashed dotted line) levels, in a single bystander H5V-Hyper cell adjacent to PI positive cells that were normalized to maximum. The Hyper low fluorescence decreased by 20%, while the Hyper-high emission elevated within 90 minutes by ∼25%.

bEnd.3 monolayers were pre-incubated with both WST11 and 2′,7′-dichlorofluorescin diacetate (DCFH-DA), a cell-permeable non-fluorescent probe (Table S1). Once in the cell, DCFH-DA undergoes de-esterification to its membrane impermeable 2′,7′-dichlorofluorescein form and remains intracellular. Oxidation modifies DCFH to its fluorescent form 2′,7′-dichlorofluorescein (DCF), which enables detection of H_2_O_2_, •OH and peroxynitrite (ONOO^−^) generation within cells [52]. Figure 3A shows a representative image from a set of experiments in which endothelial monolayers were pre-incubated with DCFH-DA, rinsed with fresh culture medium containing PI, subjected to COI and placed back into the culture incubator. Three hours later, the cells were imaged by fluorescence microscopy to detect changes in DCF fluorescence and cell death. Dead bystander cells (PI^+^), at the upper left corner, are at a distance of 50–100µm from the rim of the COI. Remarkably, live cells positioned in close proximity to the dead cell area (0–100µm) demonstrate high DCF fluorescence, indicative of intra-cellular ROS. The DCF fluorescence intensity gradually declined, reaching background levels at a distance of 500–700µm (Fig. 3B). Control cells, exposed only to laser illumination (no WST11) and imaging light to detect DCF fluorescence, show low fluorescence values, indicating minimal probe oxidation by light. Since the lifetimes of the reactive species in the biological milieu are very short (µsec to msec) [18], the fluorescence, due to intracellular ROS observed hours after COI completion, suggests continuous de-novo ROS generation in the bystander cells, far from the illuminated region, long after illumination. This conclusion was further confirmed by the negligible effect of extra-cellularly added superoxide dismutase, or catalase (Fig. 4) on the intensity and time profile of the propagation signal.

The observed ROS propagation from the COI domain could be either the cause, or the result of a bystander cell death propagation mechanism. To resolve this dilemma, we treated bEnd.3 cell monolayers with intracellular antioxidants. We anticipated that in the first case intracellular ROS quenching would result in increased cell survival, whereas in the second case such quenching would have no effect on the cell death. We chose two common cell permeable ROS scavengers, N-acetyl-L-cysteine (NAC) and vitamin C [39] to answer this question. bEnd.3 monolayers were pre-incubated with vitamin C or NAC for 1h and then rinsed free of the extra-cellular ROS scavengers. The monolayers were then subjected to COI. Subsequently, the plates were returned to the culture incubator for 3h and the extent of cell death was determined with PI and CaAMg viability probes. In both treated groups, a significant reduction in the number of dead bystander cells was observed, as compared to untreated controls (Fig. 3C). The mean bystander cell death in these groups were reduced by 73% (vitamin C) and 43% (NAC), P<0.01. In order to verify that the effect does not involve quenching of the primary COI insult, we also added vitamin C after completion of COI. This way, any decline in bystander cell death could be exclusively attributed to scavenging secondary ROS formation. The mean bystander cell death level in this group was reduced by 48% as compared to the control (Fig. 3C, P<0.01). These results strongly suggest that the observed ROS are part of the driving force of bystander death propagation, rather than passive participants in the process. Moreover, the significant response to NAC, which exerts its effect mainly by enhancing intra-cellular pools of reduced glutathione [39], depicts the involvement of intracellular ROS. The difference in cell death levels between pre and post illumination vitamin C introduction, appears therefore, to reflect primarily the contribution of the COI generated ROS.

Apparently GJIC is important for both ROS generation and injury propagation. Figure 3D, E shows that in cell cultures incubated with CBX, de-novo ROS generation was at least two folds lower (p<0.01, n = 3 including 10 cells/experiment) than in GZA treated cultures (Fig. 3E, insert). Moreover, scratching the cell monolayer prior to COI (Fig. 3F) also reduced DCF fluorescence by a factor of 2 beyond the scratch (Fig. 3F, insert). In summary, these results suggest that cell to cell contact and, more specifically, GJ coupling is required for the propagation of oxidative stress followed by death of bystander endothelial cells.

### De-novo generation of superoxide in bEnd.3 bystander cells

The most likely de-novo produced ROS in response to the COI, are O_2_^•−^, H_2_O_2_ (the latter as a product of superoxide dismutation), and •OH, that can be formed by the Fenton reaction, or decomposition of peroxynitrite [8]. Potential generators of O_2_^•−^ in cells are complex I within the mitochondria, xanthine oxidase and NADPH oxidase [53]. Several patho-physiological states were shown to activate these enzymes and, thereby, induce pathological O_2_^•−^ formation, followed by cell apoptosis [7]. Thus, we hypothesized that O_2_^•−^ is likely to be one of the ROS identified above; It was submitted for a more specific examination, using dihydroethidine (DHE), which was, converted to fluorescent 2-hydroxyethidium (and other compounds) upon oxidation by O_2_^•−^ [54], [55]. Figure 4A shows the spatial and temporal evolution of O_2_^•−^ in bystander cells post COI, at increasing distances from the activated rectangle on a time scale of hours. Cells closer to the COI rim produced O_2_^•−^ faster, to higher levels and at earlier times than more distant ones. Remote cells showed very low basal O_2_^•−^ formation. The O_2_^•−^ values directly correlate to the cell distance from the COI priming site. As noted above, the observed evolution and decay times of the O_2_^•−^ signal are orders of magnitude longer than the literature lifetimes of superoxide radicals [18]; they, therefore, cannot reflect the lag of oxygen radical diffusion from the site of primary insult. These dynamics, instead, suggest de-novo generation of O_2_^•−^ in bystander cells following the propagation of some stress signal from the COI region. Incubation with superoxide dismutase (100IU/ml) during and after COI of bEnd.3 cell cultures had no effect on the O_2_^•−^ signal formation, nor on the cell death propagation rates, reinforcing the notion that superoxide is formed intracellularly.

### De-novo generation of hydrogen peroxide (H_2_O_2_) in bEnd.3 bystander cells

The importance of H_2_O_2_ has also been recognized in both physiological and pathophysiological situations [7]. Although its life time is longer than that of a superoxide radical, its high oxidation activity renders its lifetime shorter in the biological milieu and minimizes possible migration from the photoactivation domain. Thus, H_2_O_2_ molecules can only be monitored in bystander cells a few hours post photoactivation, if generated within these cells shortly before, or during detection. To explore the contribution of H_2_O_2_ to the above DCF monitored ROS signal in bystander cells (Fig. 3), we followed fluorescence of the Hyper-H5V clone (see Materials and Methods section) after the COI. Throughout these experiments we included 100 IU/ml catalase in the culture medium to block extra-cellular generation of H_2_O_2_. Figure 4B shows Hyper-H5V fluorescence at 520nm, due to the H_2_O_2_ generation in live cells (chevrons), which precedes cell death (PI^+^, arrowheads). To quantify the HyPer signal, the ratiometric changes in the 520 nm fluorescence, were recorded upon excitation at 420 (hyper-low) and 490 nm (hyper-high), as illustrated in Fig. 4C. This plot shows TLFM of a representative bystander cell at ∼ 70µm from the rim of the COI, during 3–5 hours post photoactivation. The 520nm emission decreases overtime upon excitation at 420nm (Hyper-high), and mirrors the increase in emission upon excitation at 490nm (HyPer-high). The increase of the PI signal (signifying cell death) lags by 30 minutes after the H_2_O_2_ signal.

### De-novo generation of Peroxynitrite (ONOO^−^)

**Figure 5:**
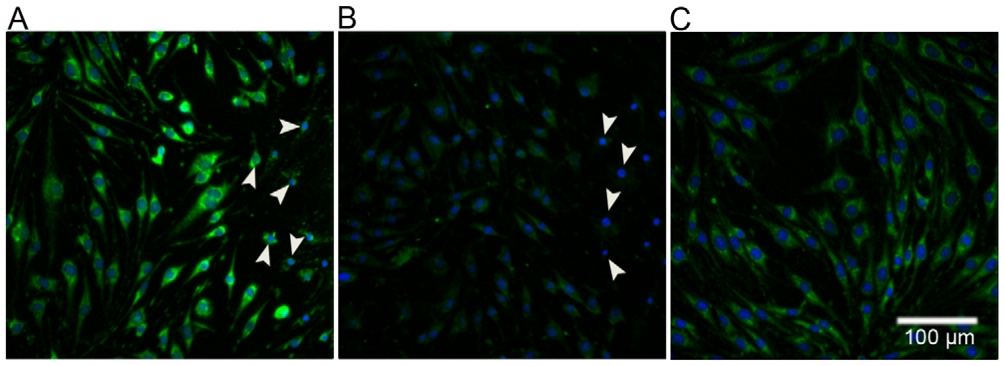
Peroxynitrite generation in bEnd.3 bystander cells following COI. **A** – Monolayers were subjected to COI, placed back in the incubator for three hours and then subjected to anti-nitrotyrosine antibodies first, and then to fluorescing secondary antibodies (pseudo-colored green). Arrowheads mark dead bystander cells (PI^+^); blue depicts DAPI nuclear stain. **B** – Control, as in (A) but with secondary antibodies only, right side arrows mark dead bystander cells. **C** – Control, untreated monolayers subjected to primary and secondary antibodies. All images represent at least three similar experiments.

ECs generate nitric oxide (NO•) following a plethora of physiological and pathophysiological situations [8], [56]. In most pathological situations, the NO• secretion coincides with the evolution of O_2_^•−^, resulting in the generation of deleterious peroxynitrite [53], known to induce endothelial dysfunction. The nitrosylation of tyrosine residues in proteins is the common footprint for the presence peroxynitrite *in-vivo* [26]. Hence, we set out to detect the evolution of peroxynitrite in bystander cells following COI by immune-staining for nitrosylated proteins. Heavy staining for nitro-tyrosine was observed in viable bystander cells adjacent to dead bystander ones three hours post COI (Fig. 5A). No nitro-tyrosine staining was observed in controls (Fig. 5B, C). These observations show that death propagation is preceded by nitrosative stress propagation, exemplified by peroxynitrite, which is most probably due to the concomitant generation of NO• and O_2_^•−^ in the bystander cells.

### Mode of bystander cell death

**Figure 6:**
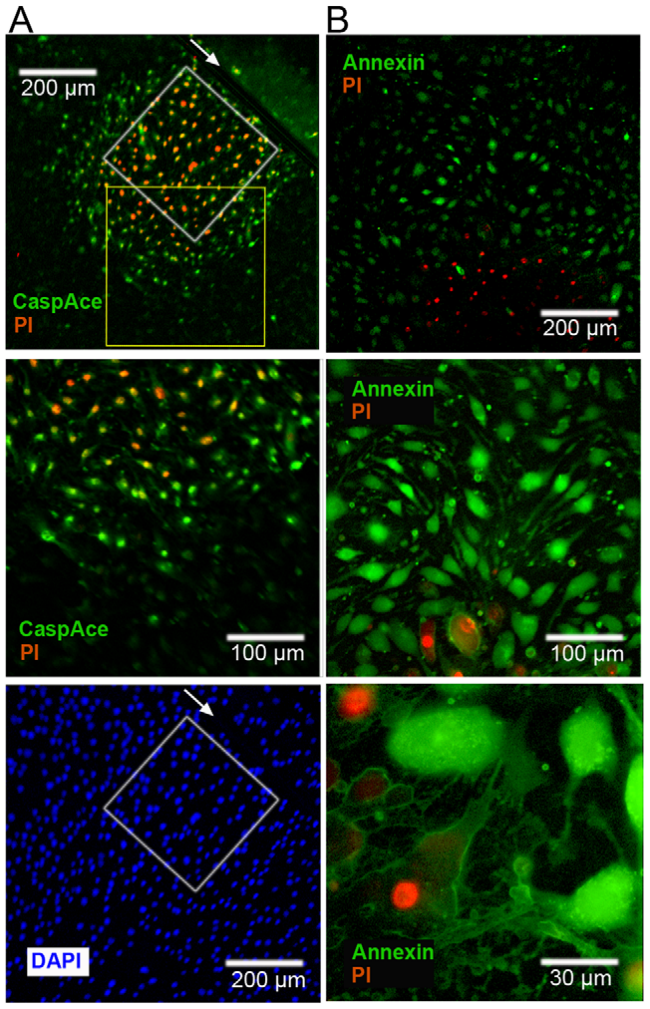
Apoptotic cell death in bystander cells following COI. **A** – bEnd.3 monolayers were linearly scratched by a surgical scalpel (arrow), subjected to COI (white rectangle, left of scratch upper panel) and placed back in the incubator for 3h. Then, CaspACE FITC-VAD-FMK, PI and DAPI were added to the cells. Upper and lower panels are snapshots of the same field of view. Middle panel is a higher magnification snapshot of the yellow rectangle in upper panel. Cell death (PI^+^) is preceded by CaspACE staining at 100–200µm beyond the COI, left of the scratch only. **B** – bEnd.3 monolayers were placed in the incubator after COI for 3h, and then subjected to Annexin-V-FITC (for phosphatidylserine depolarization) and PI. Phosphatidylserine depolarization ((Annexin-V)^+^ cells (green)) clearly precedes cell death and permeabilization (PI^+^). Panels show different magnifications. Images are a representative of n = 3 experiments.

Essentially, nitrosative or oxidative insults may result in necrotic and/or apoptosis cell death. Necrosis is characterized by initial impairment of the cell membrane integrity, enabling nuclei staining by PI. In apoptosis, the phosphatidylserine is translocated to the outer membrane, enabling Annexin-V-FITC staining; however, the membrane remains initially non-permeable to PI [57]. Thus (Annexin-V-FITC)^+^/PI^−^ suggests apoptosis. An additional critical marker is cysteine-aspartic proteases (caspase) activation. Figure 6 presents bEnd.3 cells stained for activated caspases (using CaspAce^TM^, Fig. 6A), phosphatidylserine (using Annexin-V-FITC, Fig. 6B) and cell permeabilization/death (using PI) after COI. Both CaspAce and Annexin-V-FITC staining precede PI staining by 3–4 cell rows.

### c-Jun N-terminal kinase (JNK) activation in bystander cells

**Figure 7:**
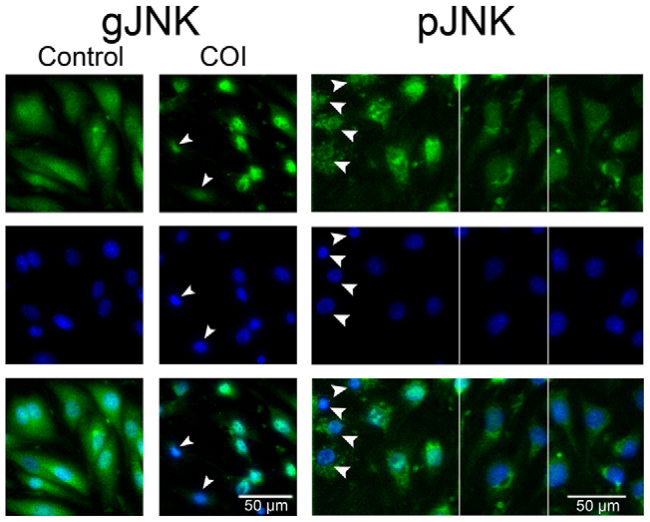
JNK activation and nuclear translocation in bystander cells. bEnd.3 monolayers, were subjected to COI or left untreated and returned into the incubator for 3 more h, then fixed with 4% PFA and stained for gJNK or pJNK (pseudo-colored green) as indicated. After immune-staining, the cell nuclei were counter stained by DAPI (pseudo-colored blue) and imaged by fluorescence microscope. gJNK: Untreated cells exhibit JNK distribution throughout the cell. In treated monolayers, bystander cells adjacent to PI^+^ cells (marked by arrowheads, 60–80µm away from rim of the COI) show distinct JNK nuclear localization. pJNK: a collage comprised of three snapshots separated by vertical white lines. Bystander cells adjacent to PI^+^ cells show pJNK in the nucleus (left side) while distant cells have no pJNK staining (right side). Lowest panels are a merge of the upper ones.

The stress-activated kinase JNK is a member of the mitogen-activated protein kinases superfamily and, upon activation, can control proliferation, differentiation and apoptosis. Intra-cellular ROS and RNS generation readily activate JNK, eventually leading to apoptotic cell death [58], [59]. Hence, we hypothesized that the apoptotic death propagation observed in the bystander ECs should be accompanied by JNK activation. To test this hypothesis, we fixed bEnd.3 monolayer cells 3h post COI and stained them by immunofluorescence for activated (phosphorylated) JNK (pJNK), or general JNK (gJNK) [42]. Figure 7 presents gJNK immunofluorescence of control, untreated cultures and displays gJNK images of bystander cells three hours post COI. Arrowheads mark dead (PI^+^) bystander cells. Untreated cells exhibit diffuse gJNK stain with no nuclear localization. Bystander cells, on the other hand, show nuclear gJNK localization, indicating their activation [60]. This nuclear translocation is observed to a distance of two cell rows from the PI^+^ bystander cells. To substantiate this observation, we immunostained pJNK under the same experimental conditions. PI^−^ pJNK^+^ bystander cells encompass two cell rows (100µm) from the PI^+^ cells, with negligible nonspecific binding and no pJNK stain beyond that point (Fig. 7). We conclude that JNK is phosphorylated and translocated into the nucleus, in response to the oxidative stress propagation. The chain of events that can lead to ROS induced JNK activation was detailed by others and involves several signaling pathways [59].

### Calcium ion involvement in bystander cell death propagation

**Figure 8:**
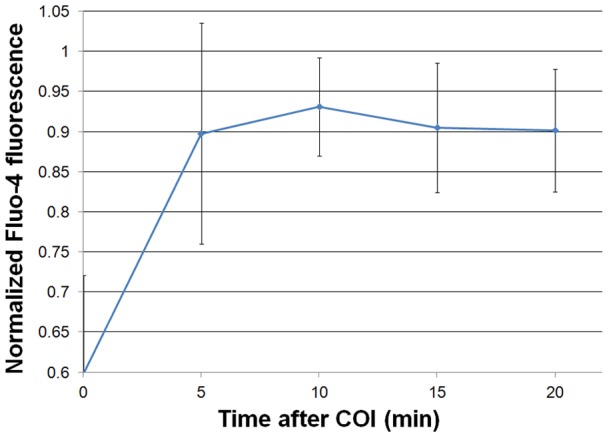
Cytosolic Ca^2+^ ions elevation in bystander cells. bEnd.3 monolayers were incubated with Fluo-4, for Ca^2+^ staining, subjected to COI and then followed by TLFM at 5min intervals. The plot illustrates the maximal normalized Fluo-4 emission intensity in n = 5 bystander cells close to the rim of the COI rectangle.

Several secondary messengers may mediate bystander cell death propagation by means of GJIC, including inositol trisphosphate, cAMP and calcium ions (Ca^2+^) [61], [62]. Recently, Ca^2+^ transients were shown during cell death propagation in a rat C6 Glioma Cytochrome-C model [40]. Changes in cytosolic Ca^2+^ may be due to transfer from adjacent cells through GJIC, or their release from intra-cellular storages by inositol trisphosphate. In a preliminary attempt to monitor such changes we used the Fluo-4 probe (which exhibits increased fluorescence intensity upon calcium binding in situ), as detailed in the Materials and Methods section. Figure 8 illustrates the normalized time dependent fluo-4 fluorescence intensity of five individual cells lying approximately 30µm from the rim of the photoactivation region. The bystander cells show sharp transients representing 20–30% elevation in Ca^2+^ levels within 5–10 minutes post COI. Approximately two hours later these cells became PI positive (not shown).

## Discussion

Understanding the chain of events leading from a locally confined oxidative insult, to a widespread tissue injury is a major challenge in basic and translational research of vascular patho-physiology. Among other means, ECs use GJIC to communicate with each other and with the myocytes surrounding them, as well as performing their physiological functions [63]. It is reasonable to assume that this communication plays a role in promoting, or possibly controlling, damage propagation from the point of injury to remote sites. Nevertheless, limited research efforts have been invested in exploring the initial response and progression modality of localized oxidative insults in the endothelium, while numerous studies focused on the myocyte response [64], [65].

In the present study, we used confluent endothelial cell cultures as a model for a living endothelium, to study its response to a spatially confined oxidative insult (COI) and the OI's spread therein. We used bEnd.3 cells, derived from primary mouse brain endothelial cells, transduced with a PymT expressing retrovirus [66]. These cells retain key features of differentiated endothelium, such as: specific protein expression (CD31, VEGF receptor 2), internalization of acetylated LDL and adhesion molecule expression, upon stimulation with pro-inflammatory cytokines [67]. The relevance of this cell line to living endothelium was further established in our lab, by demonstrating the ability of bEnd.3 cells to form 3D capillary tubes on basement membrane matrix (Fig. S2); this requires unique expression and arrangement of multiple adhesion and adherence molecules [68]. On this basis, bEnd.3 cells appear to represent an authentic model for examination of gap junction communication among endothelial cells, as also noted by others [24]. The use of these cells in our experimental setup allowed us to produce a well-defined COI and to follow the response of the entire cell population by multiple probes and tests, a task extremely challenging for *in-vivo* or *ex-vivo* studies. Nevertheless, endothelial cells *in-vivo,* interact with their surrounding vascular tissue and blood cells, and are thereby exposed to changing shear stress, as well as hemodynamic forces. As a result, they may express different connexin repertoires. Therefore, confined oxidative insults and damage propagation *in-vivo,* may also be affected by other factors, which were not addressed in our *in-vitro* model, described in the present study.

Our study shows, for the first time, that GJIC among ECs can also serve as a major route for the expansion of a localized oxidative insult. A major step towards achieving this goal was the construction of a system that enables the generation of a confined primary oxidative insult (acute local production of O2•- and •OH) to a well-defined single cell, or a small cell population, with high spatial resolution (∼7µm). This enabled, for the first time, the follow up of oxidative stress propagation from a primary insult site, in a small group of ECs, to remote sites (Figs. 1,2). By monitoring the appearance of different ROS and RNS, using several fluorescing probes, in the presence of extracellular ROS scavengers, we could differentiate between intra and extracellular reactive species, which have been recognized as important in such insults and the resulting damage propagation. It was then clearly demonstrated that a primary localized burst of oxygen radicals was followed by intercellular cues, and then mobilized through gap junctions that trigger the propagation of the oxidative assault. The ECs subjected directly to the COI appeared to undergo necrosis (rapid nuclei exposure to PI that is not preceded by phosphatidylserine translocation, Figs. 1,2). However, the bystander cells undergo apoptotic cell death following the ROS (Figs. 3 and 4) and the RNS (Fig. 5) generation, as reflected by both, phosphatidylserine translocation to the external cell membrane, and the activation of caspase-3 (Fig. 6). The two markers were examined three hours after photoactivation and were observed at a distance of 4–5 cell rows from the primary insult rim, preceding the PI staining (cell death). This observation is reinforced by the positive staining for phosphorylated and nucleus translocated JNK, a stress activated kinase [42], [58], that can lead to cell apoptosis by several mechanisms as detailed elsewhere [59]. We wish to point out that the use of several unrelated chemicals and genetically encoded ROS and RNS probes (Fig. 3, 4 and 5) significantly minimize the possibility of an experimental artifact as a cause for these results. Since bystander cell death was significantly inhibited by ROS scavengers, even when added after COI (Fig. 3), it appears that ROS play a central role in the mechanism of cell death propagation. This observation reflects the dual nature of ROS in the vascular system, acting as death inducing agents, as well as secondary messengers. Moreover, this observation may have an impact on future clinical interventions for certain vascular disorders.

The spatial spreading of newly generated ROS in the cell monolayer is restricted by their short half-life time and therefore the diffusion limits. Consequently, it is extremely unlikely that radicals formed in the primary COI will cause cell death by diffusion through GJIC, until reaching the remote death sites. However, within the current state of knowledge we cannot exclude the possibility that ROS with longer life times, such as H_2_O_2_, traverse through GJIC as secondary messengers and cause ROS generation and bystander cell death at larger distances. The identity of other potential messengers usually requires radioactive labeling of suspect compounds for their follow-up [61]. The major difficulty stems from the need to distinguish whether a molecule observed in a receiver cell is transmitted by GJIC from a donor cell or is the down-stream result of a different secondary messenger. While such investigation is beyond the scope of this study, our model can serve as a convenient platform to address it in the future.

The reported area of bystander cell death propagation in our study is markedly larger than previously reported in non-endothelial systems. Decrock and colleagues have shown the spread of apoptosis via Cx43 hemichannels after Cytochrome-C electroporation into C6 glioma cells [40]. Moreover, these authors reported apoptosis for 10–20% of the bystander cells in an area of 0–200µm from the electroporation site by six hours post cytchrome-C loading and 4–5% in the area of 200–370µm. The “apoptotic wave” shown here is far-reaching and encompasses all cells to a distance of ∼350µm from the primary square of insult. This propagation is more specific than the previously discussed release of extracellular signals (e.g. by hydrogen peroxide) that function as death signals to adjacent cells [48]. The de-novo generated peroxynitrite (the product of NO• and O_2_^•−^ interaction, Fig. 5), probably following the overwhelming activation of endothelial NADPH oxidase and eNOS [56] through the intercellular communication, may also suggest new therapeutic targets. These radicals are known to initiate apoptosis when overwhelmingly produced, as was indeed shown by our present study (Figs. 3, 4 and 5). Since GJIC also functions between ECs and myocytes [63], they may provide an interesting route to initiate damage propagation within the major components of the myovascular system, a process that might be fatal. Such a process can be mediated by Ca^2+^ mobilization from one cell to the other (endothelial bystander cells showed Ca^2+^ elevation, Fig. 8). Finally, the phosphatidylserine exposure on route of the apoptotic wave propagation is an “eat me signal” that would recruit phagocytes and, thereby, start an inflammatory process which will augment the primary oxidative damage [69].

### Conclusions

The presented findings are highly relevant to the clinical arena in several aspects:

(1) The Cx43 component that was found here to be critical for the insult and death propagation, was previously found to be up-regulated in regions of disturbed blood flow and shear-stress [24]. (2) GJIC appears to mediate other forms of damage expansion, such as the propagation of neuronal injury during ischemia-reperfusion injury [26]. (3) Studies of non-endothelial systems have suggested that the bystander effect, e.g. following deuteroporphyrin photo-activation, can be mediated by extracellular propagation of ROS [48]. However ROS, in particular O_2_^•−^ and •OH radicals, have very short lifetimes in the physiological milieu [18]; they therefore cannot migrate for more than a few microns from the site of their generation before reacting with the bio-milieu, unless undergoing a propagation mechanism, as reported for some plant systems [20]. Hence, it is extremely unlikely for this propagation to be in the form of ROS or RNS transit via GJIC.

The hereby-reported propagation of “de-novo” ROS generation utilizing GJIC enables long distance progression of an oxidative insult that augments the damage of localized vascular injury. (4) Apoptosis is an energy dependent mechanism and, therefore, expected to take place mostly in re-oxygenated, rather than ischemic tissues *in-vivo* [70]. In several animal models, massive apoptosis of cardio-myocytes is observed after blood is restored to ischemic tissue regions, thus enhancing the initial damage [71]. As noted above, the exposure of phosphatidylserine on the outer leaflet of the plasma membrane is the most universally seen alteration on the surface of apoptotic cells [72], [73]. Such exposure on ECs *in-vivo* would lead to rapid thrombus formation [74], [75] and blockade of blood flow into the acutely insulted domain. However, a wide spread of ischemic damage caused by such a response may result in organ failure, as observed in several vascular pathologies. Hence, a balance that can be assisted by therapeutic intervention, aiming at limiting the propagation of an oxidative insult, apoptotic death, and immune response, must be maintained, and may present an additional therapeutic target. Fortunately, the rate of insult propagation (in the order of hours, Figs. 1 and 2) should allow for effective intervention.

## Supporting Information

Figure S1 **Illumination region accuracy control.** Agar gel sections (3×3mm^2^) were immersed in a solution of 2mmol/L of WST11 for 1h and then left to dry overnight at 25°C. The dry gel sections were placed on a glass slide and mounted onto the microscope. WST11 fluorescence was recorded with a near infra-red filter set (Ex. 740/30nm, Em. >780nm, beam-splitter >770nm). A random field of view was chosen with the X2 objective (PlanApo 2X/0.08N.A) and a 300×300µm^2^ rectangle was assigned for laser photo-activation with the FRAPPA unit (2mW laser power, 100e3 pixel dwell time, 2 repeats, 755nm). Immediately after, the objective was replaced with the X10 (UMPlanFlN/10X/0.3 N.A 0.3) for imaging. The illuminated rectangle was recorded and averaged (panel **A**). **B** – A quantitative display of the cross section shown in **A** (white horizontal rectangle). Images are representative of n = 10 similar experiments.
(JPG)

Figure S2 **bEnd.3 cell line tube formation assay.** Basement membrane matrix (Matrigel^TM^, BD) was added to wells in a 24 well plate and gel was allowed to polymerize for 30min at 37°C. Next, a cell suspension (500µl, 40e3 cells, 5% FCS) was added to each well and the plate was placed in a humidified 37°C incubator. 24h later, tube formation was examined and imaged with a light microscope, as presented above.
(JPG)

Table S1 **Fluorescent probes used and their application details.**
(DOCX)

Methods S1 **Supporting materials about methods.**
(DOCX)

Video S1 **Time lapse fluorescence microscopy (TLFM) of endothelial cell death propagation, following confined oxidative insult (COI).** bEnd.3 cell monolayers in 3cm Petri-dishes were incubated with 20 µmol/L WST11 and viability probes (CaAMg and PI) as detailed in the materials and methods section and Table S1. Cells were subjected to COI, represented by the white 300×300µm^2^ rectangle. TLFM was launched immediately after illumination. The movie is an overlay of pseudo-colored snaps (green – CaAMg fluorescence, red – PI fluorescence). Time intervals are depicted on movie. Scale  = 100µm.
(AVI)
