# Supplementary figures and images for: Local Oxidative Stress Expansion through Endothelial Cells – A Key Role for Gap Junction Intercellular Communication
Source: PLoS One. 2012 Jul 23;7(7):e41633. doi: 10.1371/journal.pone.0041633 (PMC3402439; doi:10.1371/journal.pone.0041633)

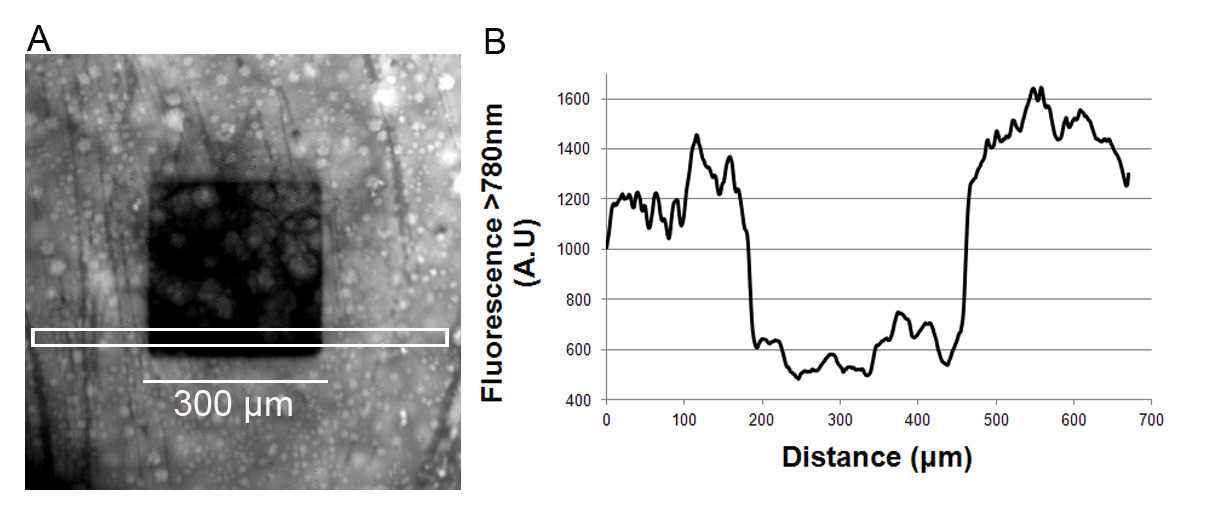

Supplement: Figure S1 — Illumination region accuracy control. Agar gel sections (3×3mm2) were immersed in a solution of 2mmol/L of WST11 for 1h and then left to dry overnight at 25°C. The dry gel sections were placed on a glass slide and mounted onto the microscope. WST11 fluorescence was recorded with a near infra-red filter set (Ex. 740/30nm, Em. >780nm, beam-splitter >770nm). A random field of view was chosen with the X2 objective (PlanApo 2X/0.08N.A) and a 300×300µm2 rectangle was assigned for laser photo-activation with the FRAPPA unit (2mW laser power, 100e3 pixel dwell time, 2 repeats, 755nm). Immediately after, the objective was replaced with the X10 (UMPlanFlN/10X/0.3 N.A 0.3) for imaging. The illuminated rectangle was recorded and averaged (panel A). B – A quantitative display of the cross section shown in A (white horizontal rectangle). Images are representative of n = 10 similar experiments. (JPG) [file pone.0041633.s001.jpg]

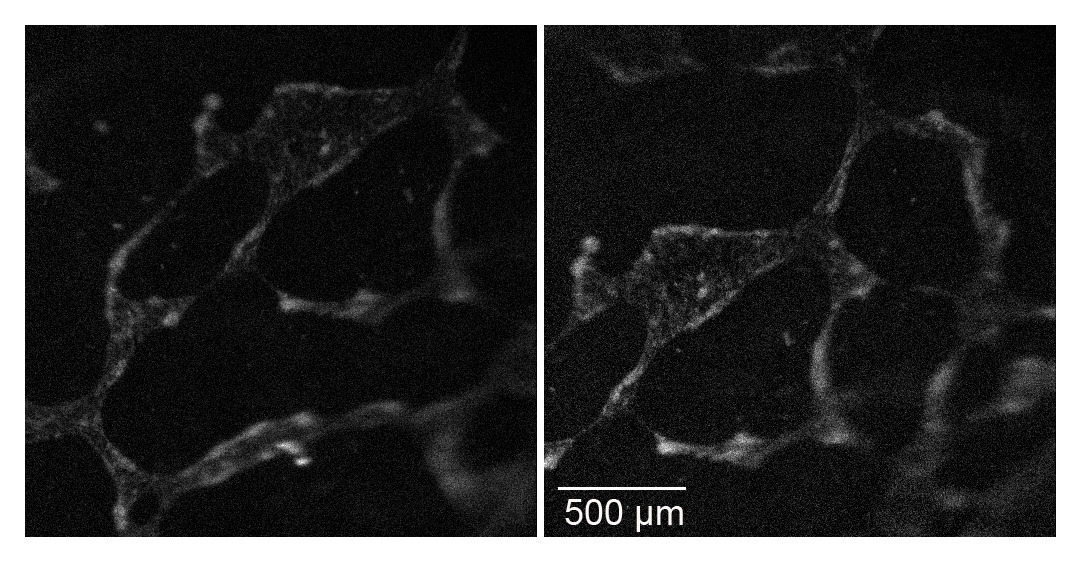

Supplement: Figure S2 — bEnd.3 cell line tube formation assay. Basement membrane matrix (MatrigelTM, BD) was added to wells in a 24 well plate and gel was allowed to polymerize for 30min at 37°C. Next, a cell suspension (500µl, 40e3 cells, 5% FCS) was added to each well and the plate was placed in a humidified 37°C incubator. 24h later, tube formation was examined and imaged with a light microscope, as presented above. (JPG) [file pone.0041633.s002.jpg]
