# Supplementary material for: Local Oxidative Stress Expansion through Endothelial Cells – A Key Role for Gap Junction Intercellular Communication
Source: PLoS One. 2012 Jul 23;7(7):e41633. doi: 10.1371/journal.pone.0041633 (PMC3402439; doi:10.1371/journal.pone.0041633)
Supplement: Table S1 — Fluorescent probes used and their application details. (DOCX) [file pone.0041633.s003.docx]

**Table S1.** Fluorescent probes used and their application details

| Probe/application | manufacturer | Final concentration | Added before/after COI (B/A) | Rinsed prior to photoactivation (Yes/No) | Incubation time (minutes) | Incubation Temperature (°C) | Excitation (nm) | Emission (nm) | Long pass filter (nm) | Reference |
| --- | --- | --- | --- | --- | --- | --- | --- | --- | --- | --- |
| DHE/superoxide detection | Molecular Probes (Gibco/Invitrogen) | 5µmol/L | B | N | 30 | 37 | 460-480 | 600-660 | 580 | [1] |
| Calcein-acetoxymethylester (calcein-AM)/viability probe | Sigma (MO, USA) | 2µmol/L | B | Y | 10 | 25 | 460-480 | 495-540 | 485 | [2] |
| Propidium Iodide (PI)/viability probe | Sigma | 2µmol/L | B | N | - | - | 535-555 | 570-625 | 565 | [2] |
| 2′,7′-Dichlorofluorescin diacetate (DCFH-DA)/intra-cellular ROS detection | Sigma | 10µmol/L | B | Y | 30 | 37 | 460-480 | 495-540 | 485 | [3] |
| Lucifer yellow/gap junction function | Sigma | 0.5mg/ml | A | - | 5 | 25 | 385-425 | 510-540 | 500 | [4] |
| CaspACE FITC-VAD-FMK/apoptotic marker | Promga (Madison, WI) | 10µmol/L | A | - | 30 | 37 | 460-480 | 495-540 | 485 | [5] |
| Annexin-V FITC/apoptotic marker | BD ( Franklin Lakes, NJ) | 5µL Annexin/100µL sample | A | - | 15 | 25 | 460-480 | 495-540 | 485 | [2] |
| DAPI/  counter stain | Molecular Probes | 100ng/ml | A | - | 5 | 25 | 325-375 | 435-485 | 400 | [6] |

References

1. Wardman P (2007) Fluorescent and luminescent probes for measurement of oxidative and nitrosative species in cells and tissues: Progress, pitfalls, and prospects. Free Radical Biology and Medicine 43: 995-1022.

2. Gatti R, Belletti S, Orlandini G, Bussolati O, Dall'Asta V, et al. (1998) Comparison of annexin V and calcein-AM as early vital markers of apoptosis in adherent cells by confocal laser microscopy. J Histochem Cytochem 46: 895-900.

3. Myhre O, Andersen J, Aarnes H, Fonnum F (2003) Evaluation of the probes 2', 7'-dichlorofluorescin diacetate, luminol, and lucigenin as indicators of reactive species formation. Biochemical pharmacology 65: 1575-1582.

4. Rozental R, Srinivas M, Spray D (2001) How to close a gap junction channel. Methods in molecular biology 154: 447–476.

5. Decrock E, De Vuyst E, Vinken M, Van Moorhem M, Vranckx K, et al. (2008) Connexin 43 hemichannels contribute to the propagation of apoptotic cell death in a rat C6 glioma cell model. Cell Death & Differentiation 16: 151-163.

6. Hunter A, Barker R, Zhu C, Gourdie R (2005) Zonula occludens-1 alters connexin43 gap junction size and organization by influencing channel accretion. Molecular biology of the cell 16: 5686.
