# Supplementary material for: Local Oxidative Stress Expansion through Endothelial Cells – A Key Role for Gap Junction Intercellular Communication
Source: PLoS One. 2012 Jul 23;7(7):e41633. doi: 10.1371/journal.pone.0041633 (PMC3402439; doi:10.1371/journal.pone.0041633)
Supplement: Methods S1 — Supporting materials about methods. (DOCX) [file pone.0041633.s004.docx]

Supporting materials about methods

Intra-cellular ROS detection

bEnd.3 monolayers were incubated with 5µmol/L DCFH-DA (Sigma, intra-cellular ROS probe) for 30min, rinsed with fresh culture medium, subjected to COI and placed back in the culture incubator for three hours. Changes in DCF fluorescence and cell death (tracked with PI) were followed by fluorescence microscopy imaging (see Table S1).

In order to determine the role of connexins in ROS propagation, carbenoxolone (CBX) a GJ uncoupler, or its’ inactive analog glycyrrhizic acid (GZA, both 100µmol/L, Sigma) were added to the plate 30 minutes prior to COI.

Superoxide detection

Intra-cellular superoxide radicals were monitored by the red fluorescence of dihydroethidine (DHE) and its oxidation product 2-hydroxyethidium [1], according to details in Table S1.

Intra-cellular hydrogen peroxide (H_2_O_2_) detection

H5V-Hyper cells encode a fluorescent cytoplasmatic fusion protein with dual excitation peaks enabling ratiometric fluorescence measurements of intracellular H_2_O_2_. For measuring intracellular generation of H_2_O_2_, COI was applied and the cell monolayers were further placed in the culture incubator for 3h prior to TLFM, using PI for cell death determination (Table S1). In these experiments 100IU/ml catalase (Sigma, bovine liver C-9322) was added to the culture medium in order to eliminate detection of extracellular H_2_O_2_.

Detection of apoptotic markers

Caspase activity was monitored by the CaspACE FITC-VAD-FMK assay kit (Promega), as previously described[2], with the following changes: bEnd.3 monolayers were subjected to COI and further incubated in the culture incubator for three hours. Next, CaspACE FITC-VAD-FMK was added to the culture medium to a final concentration of 10µmol/L together with 2µg/ml PI and kept in the incubator for 20 minutes, rinsed with PBS and incubated with DAPI in PBS for 5 minutes. Then cells were rinsed again with PBS, fixed with 4% paraform-aldehyde, sealed with a coverslip and Fluoromount-G adhesive (SouthernBiotech, Birmingham, AL) for further examination by fluorescence microscopy at wavelengths given in Table S1.

Phosphatidylserine translocation to the outer membrane was monitored by the Annexin-V-FITC assay [3] with the following changes. bEnd.3 were grown on glass coverslips, and subjected to COI and placed back into the incubator for 3h. Next, the cover slips were washed with cold PBS and immersed in 100µl Annexin binding buffer (0.1 mol/L Hepes (pH 7.4), 1.4 mol/L NaCl, 25 mmol/L CaCl_2_). Annexin-V FITC (5µl) (Molecular Probes) and 2µg/ml PI were added and the coverslip was incubated for 15 min at 25 °C in the dark. Next, the coverslip was placed in a 35mm Petri dish, 1ml of Annexin buffer was added and the stained cells were imaged by fluorescence microscopy, with wavelengths as detailed in Table S1.

References

1. Wardman P (2007) Fluorescent and luminescent probes for measurement of oxidative and nitrosative species in cells and tissues: Progress, pitfalls, and prospects. Free Radical Biology and Medicine 43: 995-1022.

2. Decrock E, De Vuyst E, Vinken M, Van Moorhem M, Vranckx K, et al. (2008) Connexin 43 hemichannels contribute to the propagation of apoptotic cell death in a rat C6 glioma cell model. Cell Death & Differentiation 16: 151-163.

3. Gatti R, Belletti S, Orlandini G, Bussolati O, Dall'Asta V, et al. (1998) Comparison of annexin V and calcein-AM as early vital markers of apoptosis in adherent cells by confocal laser microscopy. J Histochem Cytochem 46: 895-900.
